# Oral Exposure to *Phytomonas serpens* Attenuates Thrombocytopenia and Leukopenia during Acute Infection with *Trypanosoma cruzi*

**DOI:** 10.1371/journal.pone.0068299

**Published:** 2013-07-02

**Authors:** Rosiane V. da Silva, Aparecida D. Malvezi, Leonardo da Silva Augusto, Danielle Kian, Vera Lúcia H. Tatakihara, Lucy M. Yamauchi, Sueli F. Yamada-Ogatta, Luiz V. Rizzo, Sergio Schenkman, Phileno Pinge-Filho

**Affiliations:** 1 Laboratório de Imunopatologia Experimental, Departamento de Ciências Patológicas, Universidade Estadual de Londrina, Paraná, Brasil; 2 Departamento de Microbiologia, Universidade Estadual de Londrina, Paraná, Brasil; 3 Instituto Israelita de Ensino e Pesquisa Albert Einstein, São Paulo, Brasil; 4 Departamento de Microbiologia, Imunologia e Parasitologia, Universidade Federal de São Paulo, São Paulo, Brasil; Federal University of São Paulo, Brazil

## Abstract

Mice infected with *Trypanosoma cruzi*, the agent of Chagas disease, rapidly develop anemia and thrombocytopenia. These effects are partially promoted by the parasite trans-sialidase (TS), which is shed in the blood and depletes sialic acid from the platelets, inducing accelerated platelet clearance and causing thrombocytopenia during the acute phase of disease. Here, we demonstrate that oral immunization of C57BL/6 mice with *Phytomonas serpens*, a phytoflagellate parasite that shares common antigens with *T. cruzi* but has no TS activity, reduces parasite burden and prevents thrombocytopenia and leukopenia. Immunization also reduces platelet loss after intraperitoneal injection of TS. In addition, passive transfer of immune sera raised in mice against *P. serpens* prevented platelet clearance. Thus, oral exposure to *P. serpens* attenuates the progression of thrombocytopenia induced by TS from *T. cruzi*. These findings are not only important for the understanding of the pathogenesis of *T. cruzi* infection but also for developing novel approaches of intervention in Chagas disease.

## Introduction

The order Kinetoplastida is composed of flagellated unicellular organisms, some of which live in soil or aquatic environments, while others are parasites responsible for severe diseases in humans, animals, and plants [1], [2]. The combined number of people infected by Kinetoplastida pathogens is estimated to be over 20 million, resulting in various health problems and more than 100,000 deaths each year. With half a billion people at risk, mostly in tropical and subtropical areas, these parasites represent an important global health problem with associated significant economic burden [3]. Chagas disease is caused by *Trypanosoma cruzi*, a Kinetoplastid transmitted by the feces of blood-feeding triatomine insects [4]–[6]. The disease affects about 8 million people in Latin America, of whom 30–40% have or will develop neurologic manifestations [7], cardiomyopathy, and/or digestive megasyndromes [8]. The variability in disease outcome has been attributed to host responses and parasite heterogeneity [9].

*T. cruzi* infection in mice is associated with severe hematological changes, including thrombocytopenia [10], neutropenia followed by neutrophilia, and eosinophilia [11], which may contribute to mortality. Marcondes and collaborators [12] reported that acute *T. cruzi* infection is associated with anemia, thrombocytopenia, leukopenia, and bone marrow hypoplasia and that these alterations can be prevented by nifurtimox treatment. Similar hematological alterations have also been described in experimental African trypanosomiasis [13] and are common characteristics of human immunodeficiency virus infection [14] and malaria [15].

The mechanism responsible for hematological alterations observed in acute *T. cruzi* infection is not clearly understood. Our previous studies revealed that nitric oxide (NO) does not play a direct role in the development of anemia during *T. cruzi* infection, but contributes together with TNF-α to oxidative pre-hemolytic damage of erythrocytes in infected mice [16]. In addition, IFN-induced p47GTPase (LRG-47) influences *T. cruzi* control by simultaneously regulating macrophage microbicidal activity and hemopoietic function [17].

Sialic acid in the surface of *T. cruzi* plays an important role in the infectious process; however, *T. cruzi* is unable to synthesize sialic acid. Instead, the parasite expresses trans-sialidase (TS), which mediates transfer of sialic acid from host glycoconjugates to parasite mucins [18]–[20]. *T. cruzi* TS depletes platelets with sialic acid, increasing clearance and leading to thrombocytopenia during acute infection [21].

*T. cruzi* exhibit immunological cross-reactivity with other Kinetoplastida, including *Leishmania* spp. [22] and insect trypanosomatids that belong to the genera *Crithidia*, *Herpetomonas*, *Leptomonas*, and *Blastocrithidia* [23]. We showed that *Phytomonas serpens*, a tomato parasite also from the order Kinetoplastida, shares antigens with *T. cruzi* [24] but has no TS activity [25]. These antigens are recognized by human sera and induce nitric oxide-dependent protective immunity against experimental *T. cruzi* infection in susceptible BALB/c mice [24], [26], [27]. *Phytomonas* are etiologic agents of plant diseases found across southern Brazil, North and Central Africa, and several European countries. These trypanosomatids are found in plants of economic importance, including cashew, coffee, cassava, coconut, and oil palms, and infect edible fruits such as tomato, orange, guava, grape, and star fruit, [28], [29], [30]. The parasite is transmitted to plants by the bite of the coreid insect *Phthia picta,* as demonstrated by Jankevicius and collaborators using controlled cage experiments [31].

There is no information about how the protective immunity induced by *P. serpens* can modulate the biological activity of *T. cruzi* TS on thrombocytopenia and leukopenia in mice during acute *T. cruzi* infection. Here, we report that immunization with *P. serpens* prevented clearance of platelets and leukocytes from the circulation in *T. cruzi*-infected mice. Furthermore, antibodies raised by *P. serpens* immunization attenuated the thrombocytopenia induced by TS *in vivo*. Our results support the hypothesis that TS from *T. cruzi* is the causal factor of the hematological alterations observed early during infection and support the use of phytoflagellate trypanosomatids as a safer source of immunogenic agents for treatment and prevention of Chagas disease.

## Materials and Methods

### Ethics Statement

All animal procedures were performed in accordance with the guidelines of the Brazilian Code for the Use of Laboratory Animals: the protocols were approved by the Internal Scientific Commission and the Ethics in Animal Experimentation Committee of Londrina State University (Approval Number: CEEA-01.09).

### Mice

Six- to 8-week-old C57BL/6 female and male mice were supplied by the Multi-Institutional Center for Biological Investigation, State University of Campinas, Brazil. Mice were maintained under standard conditions in the animal house of the Department of Pathological Sciences, Centre for Biological Sciences, State University of Londrina. Commercial rodent diet (Nuvilab-CR1, Nuvital, Campo Mourão, Brazil) and sterilized water were available *ad libitum*. Data analysis revealed no influence of sex on experimental outcomes.

### Parasites

*T. cruzi* Y [32], a generous gift from Dr. Paulo Araújo, State University of Campinas, Brazil, was maintained by weekly intraperitoneal (i.p.) inoculation of Swiss mice with 2×10^5^ trypomastigotes. To conduct our experiments, blood from previously inoculated Swiss mice was obtained by cardiac puncture with heparinized syringes.

*P. serpens* 15 T (see Figure S1) isolated from tomato fruit (*Lycopersicum esculentum*) [31] was cultured in GYPMI medium (glucose, yeast extract, peptone, and meat infusion) [24] at 28°C.

### Immunization of Mice and Challenge with *T. cruzi*

For immunization of C57BL/6, living forms of *P. serpens* 15 T collected during log phase growth [31] were washed 3 times by centrifugation at 3000 g for 5 min in 15 mM PBS (phosphate-buffered saline, pH 7.2) and administered by gavage (per os). Each inoculum consisted of 2×10^8^ living parasites/0.1 mL in 15 mM PBS, pH 7.2 given 4 times at 1-week intervals [24]. Seven days after the last oral immunization with *P. serpens*, C57BL/6 mice were infected i.p. with a non-lethal (10^2^ or 5×10^3^ cells/animal) or lethal (5×10^5^ cells/animal) doses of trypomastigotes. Control mice received PBS alone.

### Hematological Methods

Peripheral blood was collected from uninfected and infected mice by cardiac puncture under ether anesthesia and counted by standard methods [33]. Platelets were counted in peripheral blood collected in polypropylene tubes containing 3.8% (w/v) sodium citrate (citrate: blood ratio, 1∶9) [21]. All manipulations were carried out at room temperature. Platelets and leukocytes were counted manually with a Neubauer hemocytometer. All blood analysis and cell counts were performed 7, 12, or 21 days post-infection (p.i.).

### Bone Marrow Cell Harvest

Bone marrow cells were harvested by flushing the femoral shafts with ice-cold PBS. The total number of megakaryocytes in cell suspensions from uninfected and infected mice (12 days p.i.) was determined by hemocytometer counting [12], [34].

### Monoclonal Antibody Anti-TS (mAb 39)

mAb 39 was selected from hybridomas prepared by fusion of spleen mice immunized with membrane fraction of *T. cruzi* trypomastigotes and p3U1 cells [36]. Antigen was prepared by three cycles of freeze-thawing trypomastigotes in detergent-free buffer and supernatant collection after centrifugation at 100,000 g. Positive clones were screened by immunoblotting of total trypomastigote lysates in SDS-PAGE sample buffer. Positive clones were further cloned by limiting dilution and injected i.p. into mice primed 24 h before with incomplete Freund Adjuvant. Ascitic fluids were collected. Alternatively, antibodies were purified by affinity chromatography with protein A Sepharose (GE) following standard procedures.

### Trans-sialidase (TS) Production and Purification

Recombinant *T. cruzi* TS lacking the carboxy-terminal repeats was purified from *Escherichia coli* BL21 DE3 pLysS. The construct was ligated into the *Nde*I and *Bam*HI sites of pET 14b (Novagen) using a fragment derived from pTS16 as described by Schenkman and collaborators [35]. Expression was induced with 0.1 mM IPTG for 20 h at 28°C. Cells collected by centrifugation, washed in 20 mM Tris-HCl pH 8, resuspended in the same buffer containing 0.1 M NaCl and a cocktail of protease inhibitors (EDTA-free, Roche), and lysed by 3 passages in a French Press. The extract was clarified by centrifugation at 10,000 g (30 min) and separated on a Ni2+-agarose column (Qiagen). Unbound material was washed with 100 volumes of 0.1 M NaCl, 50 mM sodium phosphate, pH 8 and 10 mL of the same buffer, but at pH 6, and eluted from the column in the same buffer containing 0.25 M imidazole. The eluted material was dialyzed against 20 mM Tris-HCl pH 8 and separated on a MonoQ column. Fractions containing purified enzyme, as judged by SDS-PAGE, were eluted between 0.15 and 0.2 M NaCl and stored at 4°C until use. Native TS was purified from LLC-MK2 culture supernatants by affinity chromatography on mAb 39 immobilized on CNBr-Sepharose, as described previously [36].

### Mouse Immune Sera

Ten C57BL/6 male mice were treated with 2×10^8^ living culture of *P. serpens* (15 T strain) by gavage 4 times at 7-day intervals. One week after the last inoculum, sera from immunized mice were collected and used as hyperimmune sera against *P. serpens*. All sera were tested by direct agglutination for *P. serpens* as described previously [24].

### TS Administration in Mice

Animals were injected i.p. with 50 µg enzyme in 0.1 mL PBS for each experiment. We used a dose that is five times higher as used in reference [21] because it was injected intraperitoneally. Control mice received PBS alone. In 2 separate experiments, mice received a single dose (0.1 mL) of immune sera anti-*P. serpens* mixed with purified TS (50 µg) for 15 min at room temperature. Serum samples obtained from naïve mice were used as controls. Twenty-four hours after injection, platelets and leukocytes were counted.

### Immunoblotting and ELISA

Parasite protein lysates were analyzed by SDS-PAGE. Resolved proteins were electrophoretically transferred to nitrocellulose membranes (Hybond C, Amersham Biosciences, England) for western blotting. Membranes were blocked in 5% skim milk in PBS for 16 h at 4°C or 2 h at 22°C. After washing with PBS/0.2% Tween 20, membranes were incubated with diluted antibodies for 2 h at room temperature, then washed and incubated with peroxidase-conjugated goat anti-rabbit or anti-mouse secondary antibody (Sigma, 1∶10000) for 1 h. Detection was performed according to the manufacturer’s instructions. For ELISA, 96-well plates were coated by incubating 50 µL native TS at 10 µg/mL in 0.1 M sodium bicarbonate (pH 8.5) for 12 h at 4°C. The protein was removed and wells washed 3 times in PBS containing 0.05% Tween 20 and incubated in the same buffer with 2% BSA and 2.5% skim milk for 1 h at 25°C. Antibodies diluted in blocking solution were incubated 1 h at 25°C, washed 5 times with PBS, 0.05% Tween 20, and detected as described above using *o*-phenylenediamine and H_2_O_2_.

### Statistical Analysis

Statistical analysis was conducted using ANOVA with the Bonferroni test. Comparisons between experimental groups were performed using Student’s *t*-test. Values are presented as mean ± SE. Differences were considered significant when *p*<0.05.

## Results

### *T. cruzi*-induced Transient Thrombocytopenia and Leukopenia

**Figure 1:**
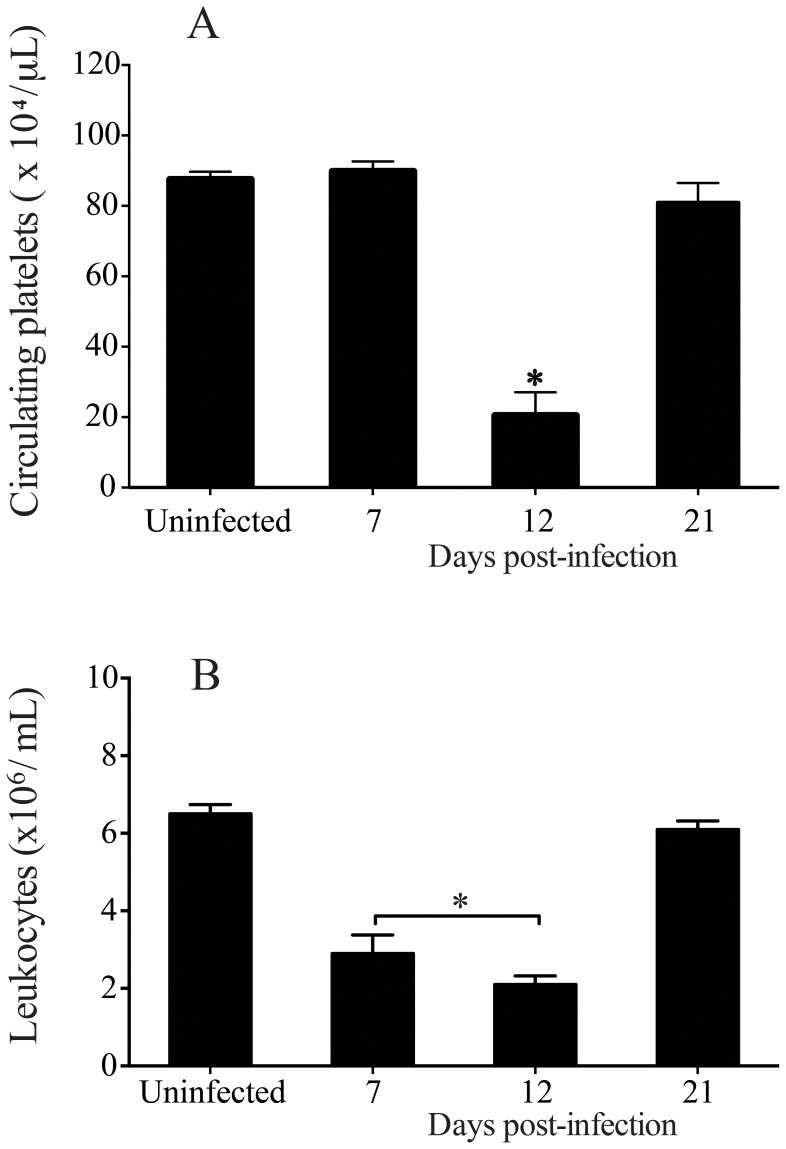
*T.* *cruzi* infection induces thrombocytopenia and leukopenia transients in the early of infection. C5BL/6 mice were infected with 5×10^3^ trypomastigotes (Y strain) via i.p. injection, monitored for the development of thrombocytopenia and leukopenia, and sacrificed at different time points post-*T. cruzi* infection. *A*: platelets and *B*: leukocytes were counts from peripheral blood from uninfected and infected mice. Values represent the mean ± standard error and are representative of three independent experiments, using 4–15 mice per group. Results were analyzed by analysis of variance (ANOVA) followed by Bonferroni multiple comparisons test. Asterisks indicates significant differences (*p<*0.05) when compared with control group (uninfected).

**Figure 2:**
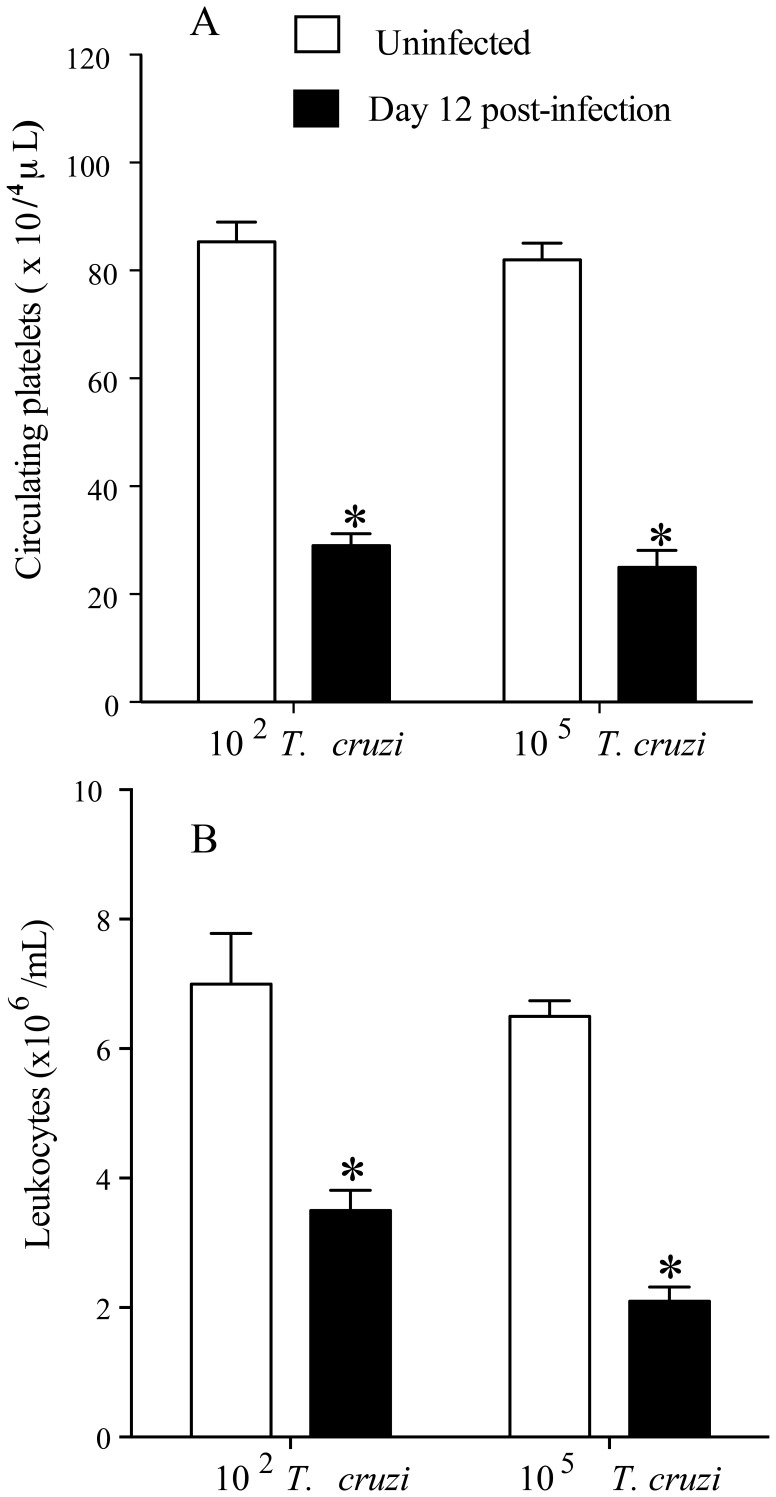
*T.* *cruzi* infection induces thrombocytopenia and leukopenia independent of the number of parasites used for infection. Groups of C5BL/6 mice were infected with 10^2^ or with 10^5^ trypomastigotes (Y strain). *A*: platelets and *B:* leukocytes were counts from peripheral blood from uninfected and infected mice. All cell counts were performed 12 days p.i. Values represent the mean ± standard error and are representative of two independent experiments, using 6 mice per group. Comparisons between 2 experimental groups were performed using Student’s *t*-test. Asterisks indicate significant differences (*p*≤0.001) when compared with control group (uninfected).

We initially confirmed that *T. cruzi* infection caused transient changes in platelets and leukocytes counts. As shown in figure 1, C57BL/6 mice infected with 5×10^3^ blood *T. cruzi* trypomastigotes developed thrombocytopenia (Figure 1A) and leukopenia (Figure 1B) during the early stages of infection (day 12 p.i.), a transient effect that lasted as long as acute phase of *T. cruzi* infection remained. Platelet counts started to decrease after seven days of *T. cruzi* infection (day 8 p.i = 67.5±6.6×10^4^/µL) and values returned to normal by day 35 p.i (103.3±11.7×10^4^/µL) (data not shown). Interestingly, *T. cruzi*-infected mice developed thrombocytopenia (day 12 p.i) when using different inoculum of parasites (Figures 2 A and 2 B).

### Oral Immunization with *P. serpens* Prevented Reduction of Platelets and Leukocytes

**Figure 3:**
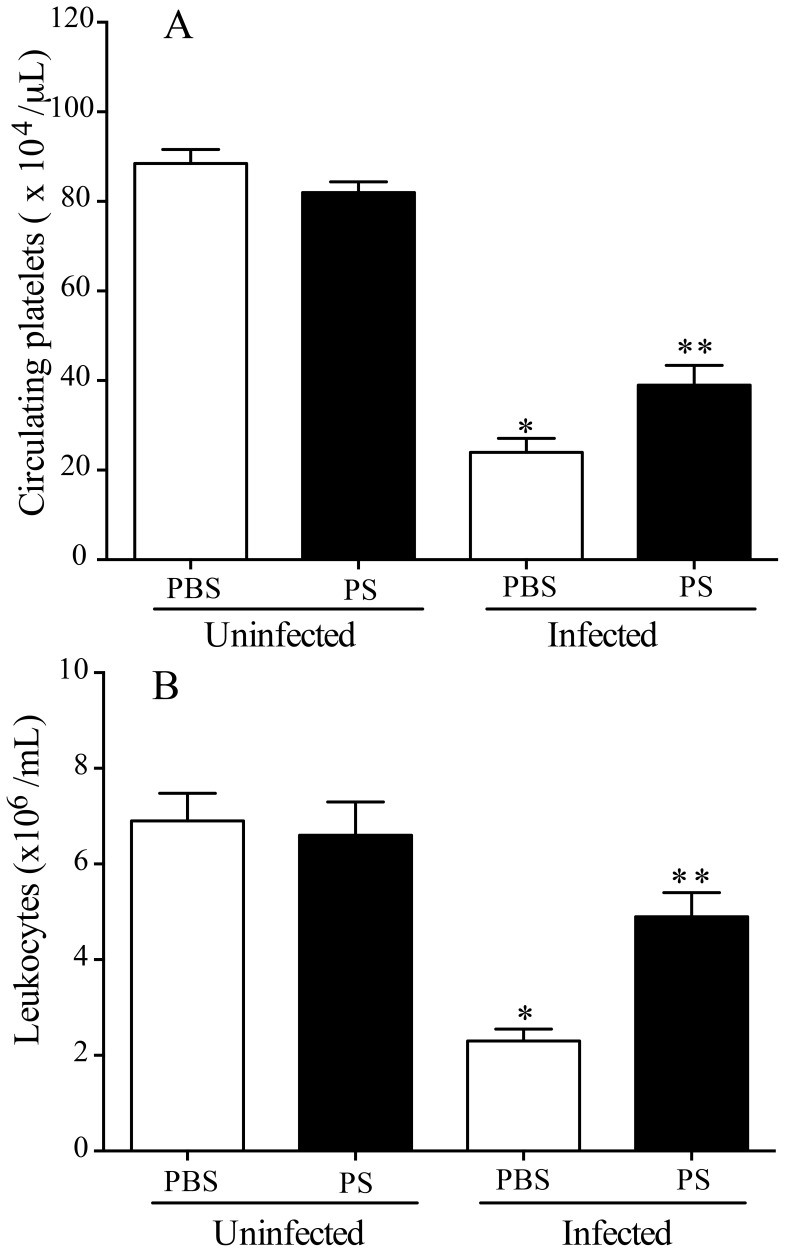
Oral exposure to *P.* *serpens* attenuates thrombocytopenia and leukopenia induced by *T. cruzi* infection. C57BL/6 mice received by gavage 2×10^8^ living *P. serpens* parasites four times at weekly intervals and an i.p. challenge 1 week later with 10^5^ blood trypomastigotes by i.p. route. Whole blood samples were collected on day 12 p.i. *A*: Platelets counts and *B*: Leukocytes counts. Values represent the mean ± standard error and are representative of three independent experiments, using 8–12 mice per group. Results were analyzed by analysis of variance (ANOVA) followed by Bonferroni multiple comparisons test. Asterisks indicate significant differences (*p<*0.001) between infected and uninfected controls. Double asterisks indicate significant differences (*p<*0.05) between infected mice given PBS (phosphate-buffered saline, pH 7.2) or immunized with *P. serpens* (PS) prior to infection with *T. cruzi*.

**Figure 4:**
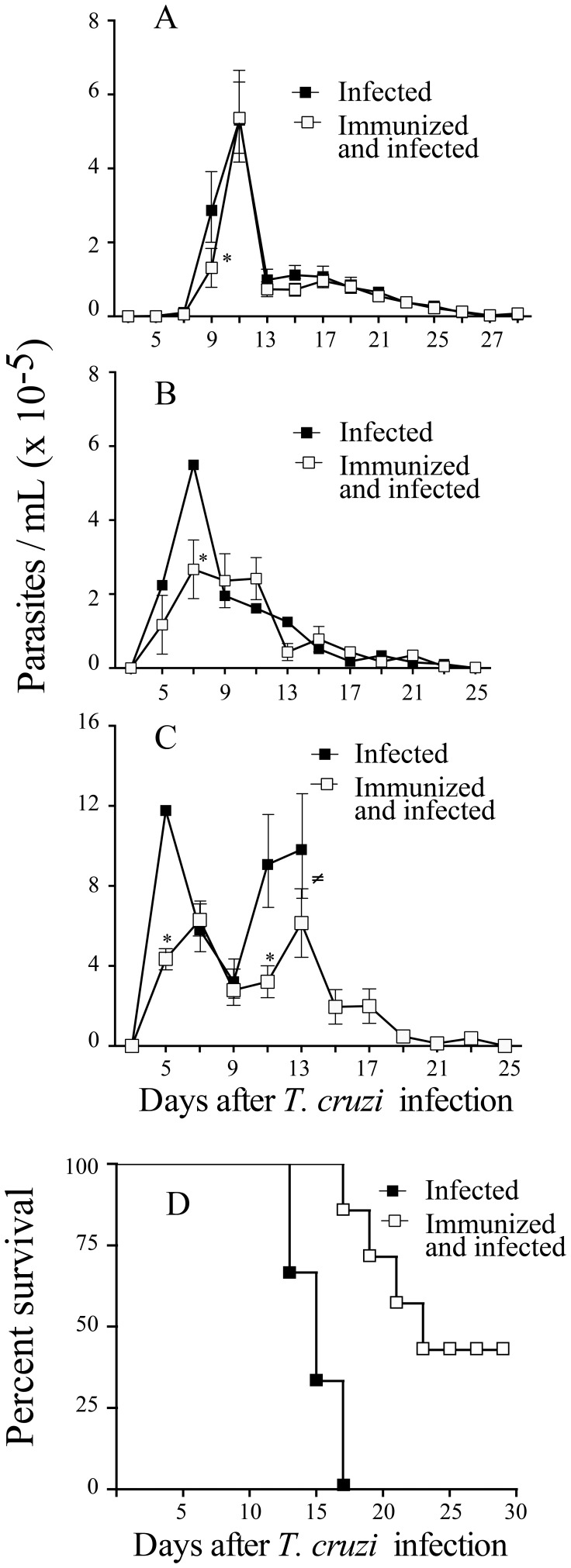
Oral exposure to *P.* *serpens* decreases parasitemia and mortality in response to *T. cruzi* infection. C57BL/6 mice were immunized with *P. serpens* (2×10^8^ living parasites per 0.1 mL PBS administered by gavage) four times with one-week intervals. Seven days after the last immunization mice were infected with Y strain of *T. cruzi*, *A*: 10^2^, *B*: 5×10^3^ and *C*: 10^5^ trypomastigote forms, respectively. Parasitemia were assessed over 30 days post infection. D: Survival of immunized mice and infected with 10^5^ (lethal dose). Data are represented as mean ± standard error represented of at last 10 mice per group. Asterisks indicates significant differences (*p*<0.05). (≠) indicates that all animals died.

Next, we tested whether immunization with *P. serpens* prevent reduction in vivo of platelets and leukocytes in the host infected with *T. cruzi*. For this, C57BL/6 mice previously immunized were infected with trypomastigote forms of *T. cruzi* (10^5^ cells/mouse, lethal dose). Twelve days after infection, immunized mice displayed a reduced decrease in platelets and leukocytes counts (Figures 3 A and 3 B, *p = *0.001) when compared with control mice. Thus, platelets and leukocytes in infected mice appear to be sensitive to oral exposure to *P. serpens*. Moreover, the immunization reduced the parasitemia upon a *T. cruzi* challenge (Figure 4, *p<*0.05). This reduction occurs in the early of infection and protected mice to lethal dose of parasites (Figure 4C and Figure 4D, *p<*0.05).

### TS Reduces Platelet Blood Counts

**Figure 5:**
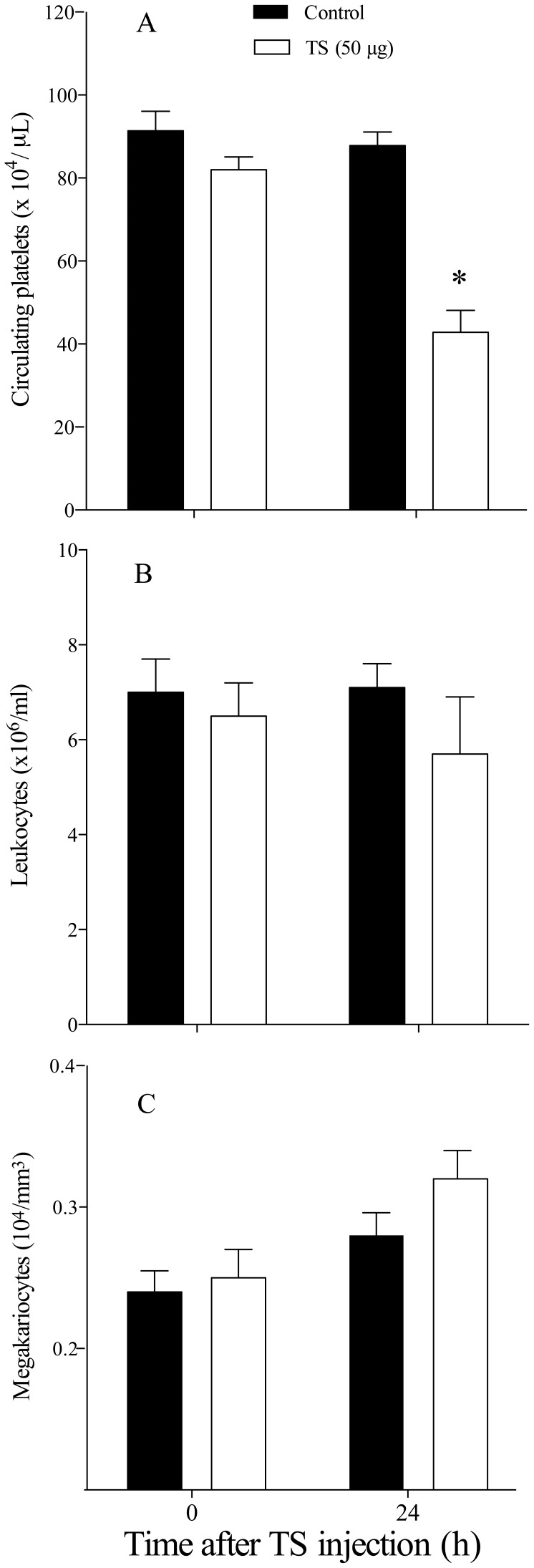
TS reduces blood platelets counts in naïve C57BL/6 mice. Mice were inoculated i.p with 50 µg of recombinant TS. *A*: platelet, *B*: leukocyte and *C*: megakariocyte counts were determined 24 h later. Values represent the mean ± standard error and are representative of two independent experiments; using 7–15 mice per group Results were analyzed by analysis of variance (ANOVA) followed by Bonferroni multiple comparisons test. Asterisks indicate significant differences (*p*<0.05).

When TS (50 µg) was injected intraperitonially in mice (same route of *T. cruzi* infection), a strong reduction (around 50%) in the normal platelet count was observed 24 h after the enzyme injection (Figure 5 A, *p<*0.05). No effect was observed when TS was heat-inactivated (see Figure S2). We did not observe changes in the leukocyte (Figure 5B) and megakaryocytes (Figure 5C) counts of mice inoculated with TS.

### Oral Exposure to *P. serpens* Reduces Thrombocytopenia Induced by TS

**Figure 6:**
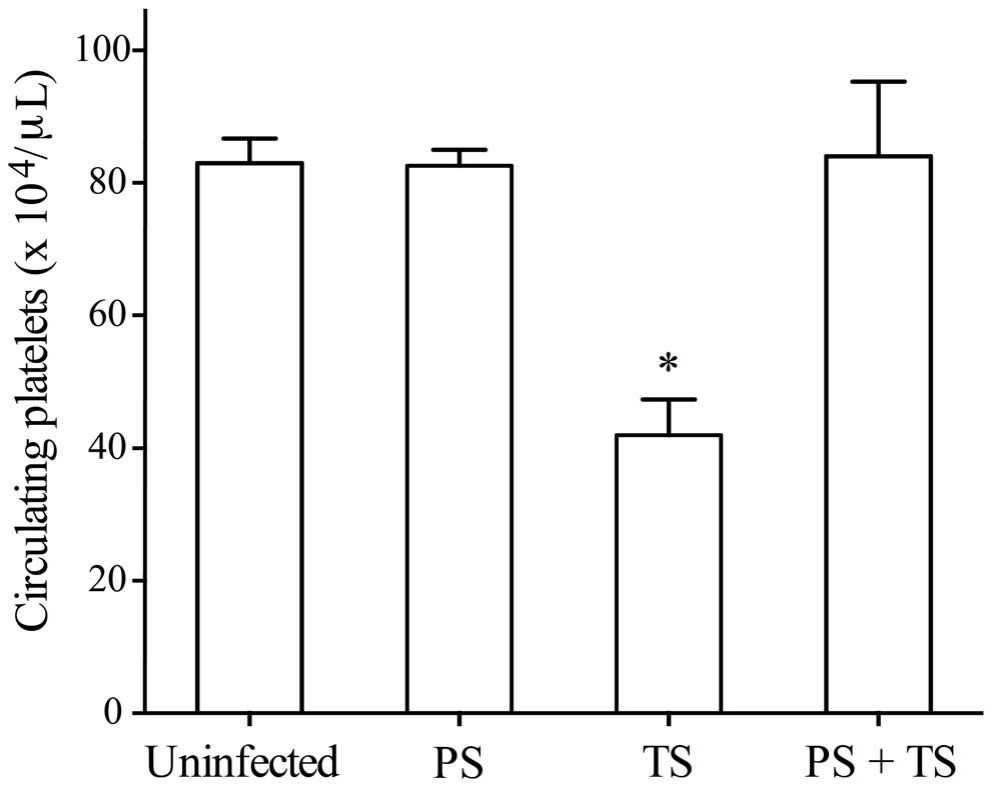
Oral exposure to *P.* *serpens* restored the thrombocytopenia induced by TS. C57BL/6 Mice received by gavage 2×10^8^ living *P. serpens* parasites four times at weekly intervals and an i.p. challenge 1 week the mice were inoculated i.p with 50 µg of recombinant TS. Values represent the mean ± standard error and are representative of two independent experiments, using 7–10 mice per group. Results were analyzed by analysis of variance (ANOVA) followed by Bonferroni multiple comparisons test. PBS (phosphate-buffered saline, pH 7.2) and PS (immunized with *P. serpens*). Asterisks indicate significant differences (*p*≤0.001) when compared with control group (uninfected).

To test whether oral immunization with *P. serpens* was able to reduce the effects of TS on platelets, the enzyme was directly injected in immunized animals. As shown in Figure 6, the immunization prevented the effects of TS on platelets in mice. Furthermore, the counts of megakaryocytes in the bone marrow were essentially the same in all groups (Figure S3, *p>*0.05).

### Hyperimmune Sera Against *P. serpens* Attenuates the Effects of TS on Platelet Counts

**Figure 7:**
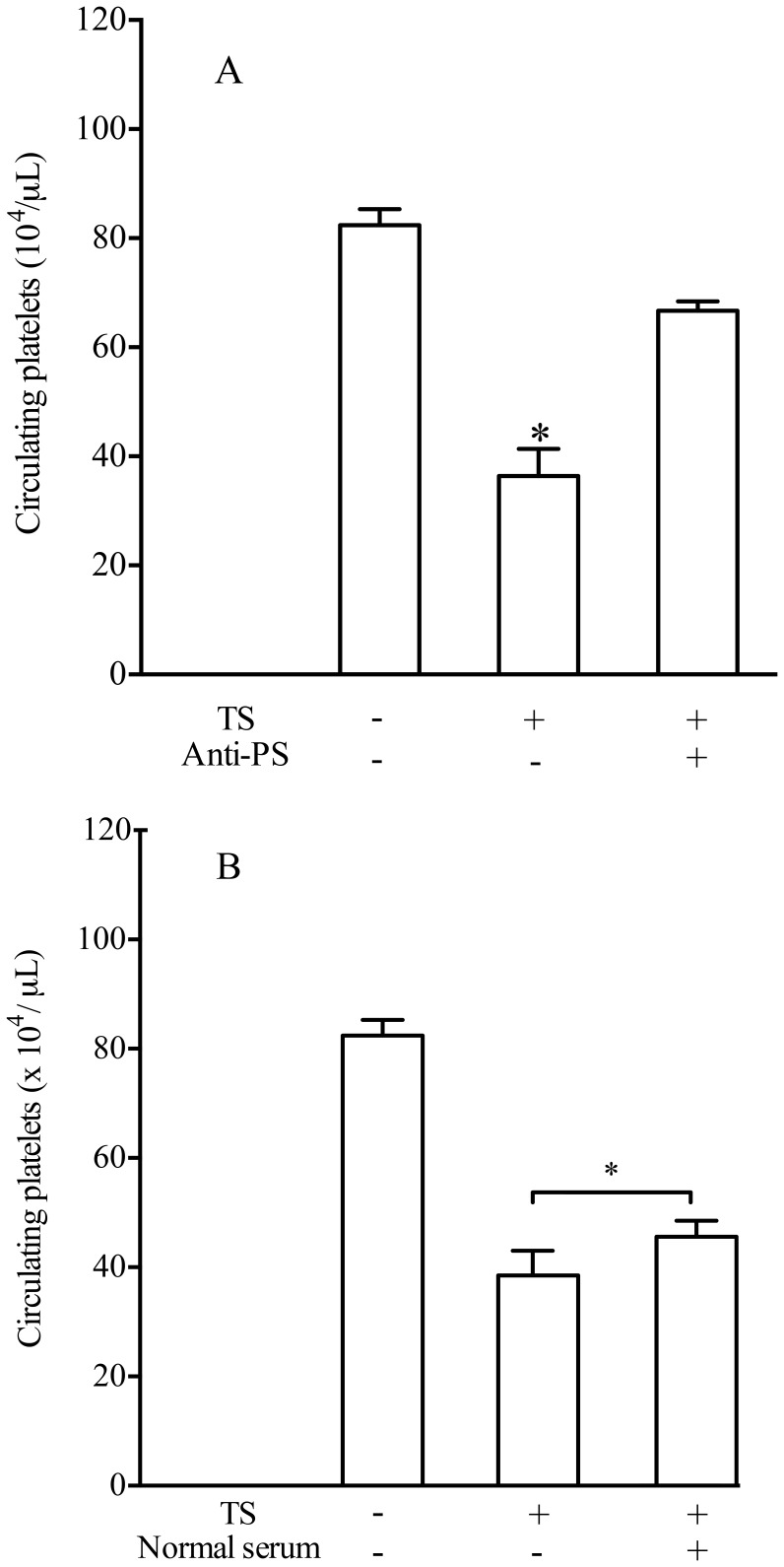
Sera of *P. serpens*-immunized mice inhibit the activity of TS on platelets. The mice were injected i.p. with 50 µg of TS in 0.1 ml previously mixed with immune serum A or with normal serum B. A third group of mice received only PBS instead of TS. Platelet counts were determined 24 h later. Values represent the mean ± standard error and are representative of two independent experiments, using 4–7 mice per group. Results were analyzed by analysis of variance (ANOVA) followed by Bonferroni multiple comparisons test. Asterisks indicate significant differences (*p*<0.05) of samples compared with PBS.

**Figure 8:**
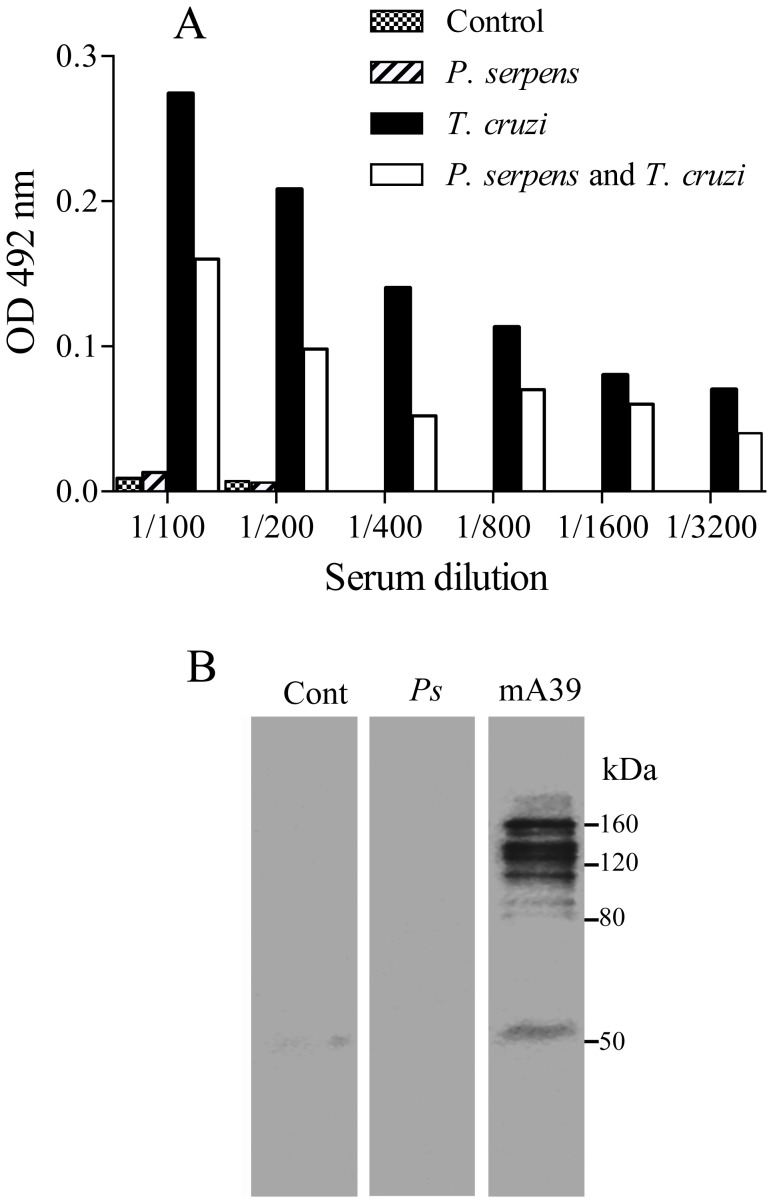
Mice immunized with *P.* *serpens* lack antibodies to TS. *A*. ELISA of plates coated with recombinant TS using control mice serum or sera immunized with *P. serpens*, followed by infection with *T. cruzi. B*. Immunoblotting showing the polypeptides recognized by hyperimmune sera anti-*P. serpens* detected in the whole cellular extract from *P. serpens*. Alternatively, the TS was also revealed using anti-TS antibody (mAb 39). Number on the left indicate the apparent molecular mass of protein standards expressed in kDa.

Incubation of sera from mice immunized with *P. serpens* with TS inhibited its thrombocytopenic effect on platelets (Figure 7 A, *p*<0.05). Moreover, serum samples obtained from naïve mice do not inhibit the action of TS *in vivo* (Figure 7 B, *p>*0.05). By means of immunoblotting analyses we showed that antibodies present in the serum of *P. serpens*-immunized mice recognized polypeptides in the cellular extract of *P. serpens*. However, antibodies from mice immunized with *P. serpens* did not recognize the purified native TS, which was recognized by the monoclonal antibody anti-TS (mAb 39) in Western blots (Figure 8). Indeed, no effect of anti-*P. serpens* sera in the enzymatic activity was found using sialyllactose as substrates (data not shown).

## Discussion

During the experimental infection of mice with *T. cruzi*, several hematological abnormal parameters, including marked thrombocytopenia and leukopenia, are observed [11], [13], [16], [17], [37]. These alterations are transient [10] and can be prevented by trypanocidal drugs [12], but there is still no suitable molecular explanation for this effect. By using a model of infection with a *T. cruzi* Y strain in C57BL/6 and BALB/c mice, which are prototype hosts for the study of resistance and anemia in murine Chagas disease [34], we could obtain in C57BL/6 mice (resistant type) a more severe anemia compared to Swiss (susceptible mice) [37]. Ours results demonstrate for the first time, that the previous immunization with *P. serpens*, a tomato parasite, prevented the clearance of platelets and leukocytes from circulation in *T. cruzi*-infected mice. In addition, we found that a single i.p. injection (same route of infection with *T. cruzi*) of TS into mice reduced the platelet count by 50%, 24 h after TS injection. As described previously, TS has the ability to disseminate systemically within the host: the blood and bone marrow are the main sites where the enzyme may act to reduce the platelet count [21]. More important, the immunization with *P. serpens* reverted the effects of TS on platelets in mice. We did not observe changes in the leukocyte and megakaryocytes counts from mice inoculated with TS and previously immunized. These data could indicate that TS does not cause deleterious effects on the bone marrow and confirms the studies of Tribulatti and co-workers [21].

*T. cruzi* is unable to synthesize sialic acids de novo [38], but circumvents this limitation by expressing the enzyme TS, which is able to directly transfer α(2, 3)-linked sialyl residues among the glycoproteins or glycolipids [20]. TS is anchored to the membrane by glycosylphosphatidylinositol and is shed into the surrounding environment, and it is detected in the blood of infected animals and human patients during the acute stage of the infection [39]–[41]. In the C-terminus, the enzyme has tandem repetitive amino acid units that allow it to persist in blood for at least 3 days [42], allowing it to induce pathological disorders even far from the infectious foci or to act on the blood cells [39]. Previous reports indicated that TS from *T. cruzi* alters the platelet surface sialic acid content, acting as a neuraminidase [39], which induces accelerated clearance of the platelets leading to the thrombocytopenia observed during acute Chagas disease [21]. Also, immunization with culture forms of insect trypanosomatids has been shown to induce partial protection against lethal *T. cruzi* inoculations [43]. In fact, we previously demonstrated that BALB/c mice immunized with *P. serpens* and later challenged with a lethal inoculum of *T. cruzi* trypomastigote forms show a significant decrease in parasitemia and mortality [24]. As described previously, *P. serpens* is highly immunogenic in mice and rabbits [24] and the sera from patients with Chagas disease present a strong reactivity to *P. serpens* antigens [26], [44].

The results showing that hyperimmune sera against *P. serpens* attenuated the effect of TS on platelets led us to ask whether this inhibition was due to the presence of specific antibodies directed against the enzymatic domain of TS since *P. serpens* shares common antigens with *T. cruzi* [26], [45]. However, the serum from mice immunized with *P. serpens* do not recognize TS when tested by Western blot or ELISA. Moreover, the monoclonal antibody anti-TS (mAb 39) [36] do not recognize antigens in *P. serpens.* In addition, no effect of sera in the enzymatic activity was found using sialyllactose as substrates, while inhibit of platelet desialylation was previously observed. These results suggest that the effect of *P. serpens* immunization could be done through recognizing or changing the substrate *in vivo*. Alternatively, the inhibition could be consequence of changes in immune responses, as upon *T. cruzi* infection TS elicits the formation of antibodies that inhibit its activity (also called neutralizing and anti-catalytic domain), and non-inhibitory antibodies (anti-lectin-like, anti-SAPA, and anti-several epitopes on the proteins), also called cross-reacting determinants [20]. Because of the cross reactive determinants shared by TS with *T. cruzi* other molecules, v.g. SAPA, mainly situated on the non-catalytic portion of the TS, it is conceivable that these highly conserved sequences are present also in *P. serpens*. Indeed, sialidases are highly conserved in insect trypanosomatids and also in some bacteria [46]. Therefore, antibodies to *P. serpens* cross-reactive to the non-catalytic portion of TS could neutralize the injected TS, which would prevent its activity on the platelets. However, the frequency and of such antibodies in the polyclonal *P. serpens* immune serum could be low and prevent their direct detection. We confirmed the presence of anti-TS antibodies on day 12 after infection, which might contribute to decrease the thrombocytopenia. For example, mice infected with *T. cruzi* produce antibodies that are able to neutralize TS activity only if mice survive the acute phase of infection [40]. Accordingly, the sera from chronic Chagasic patients and rodents infected with *T. cruzi* can inhibit TS by recognizing its amino-terminal and catalytic domain [41]. Another possibility is that hyperimmune *P. serpens* sera affect TS clearance preventing platelet desialylation. Moreover, the reduction in the clearance of platelets from circulation in *T. cruzi*-infected mice and previously immunized with *P. serpens*, can be partly explained if we consider a wide distribution of the carbohydrate epitopes galactosyl α(1–3) galactose in *P. serpens*, as described by Gazzinelli and collaborators [47]. In fact, potent inhibitors of *T. cruzi* propagation *in vitro* and *in vivo* IN humans are antibodies directed against TS or the α-galactosyl residues of trypanosomal mucins [48]. Therefore, antibodies to carbohydrate epitopes present in the sera of animals immunized with *P. serpens* could promote a decrease of activity of TS on platelets.

In conclusion, our observations demonstrate an effect of *P. serpens* immunization of the action of TS on platelets during *T. cruzi* infection. Further elucidation of the mechanism by which the *P. serpens* affect TS can provide new tools to understand the progression of Chagas disease.

## Supporting Information

Figure S1 **Tomatoes (** ***Lycopersicum esculentum*** **) infected with** ***Phytomonas serpens*** **.** ***A and B*** **.** (**A**) Tomatoes infected. (**B**) Living culture flagellates forms of *P. serpens* (original magnification 400 X). Arrows indicate local infection on the fruit.
(TIF)

Figure S2 **Heat-inactivated TS does not modify the life span of platelets.** C57BL/6 mice were inoculated i.p. with 50 µg of recombinant TS heat-inactivated. Platelets counts were determined 24 h later. Values represent the mean ± standard error and are representative of two independent experiments; using 4 mice per group Results were analyzed by analysis of variance (ANOVA) followed by Bonferroni multiple comparisons test. Asterisks indicate significant differences (*p*<0.05) when compared with control group (PBS).
(TIF)

Figure S3 **Oral exposure to** ***P. serpens*** **does not induce alterations in megakariocyte counts.** The mice received by gavage 2×10^8^ living *P. serpens* parasites four times at weekly intervals and an i.p. 1 week later the mice were inoculated i.p. with 50 µg of recombinant TS. Megakariocyte counts were determined 24 h later. Values represent the mean ± standard error and are representative of two independent experiments, using 12 mice per group. Results were analyzed by analysis of variance (ANOVA) followed by Bonferroni multiple comparisons test. PBS (phosphate-buffered saline, pH 7.2) and PS (immunized with *P. serpens*).
(TIF)
