# Supplementary figures and images for: Oral Exposure to Phytomonas serpens Attenuates Thrombocytopenia and Leukopenia during Acute Infection with Trypanosoma cruzi
Source: PLoS One. 2013 Jul 2;8(7):e68299. doi: 10.1371/journal.pone.0068299 (PMC3699546; doi:10.1371/journal.pone.0068299)

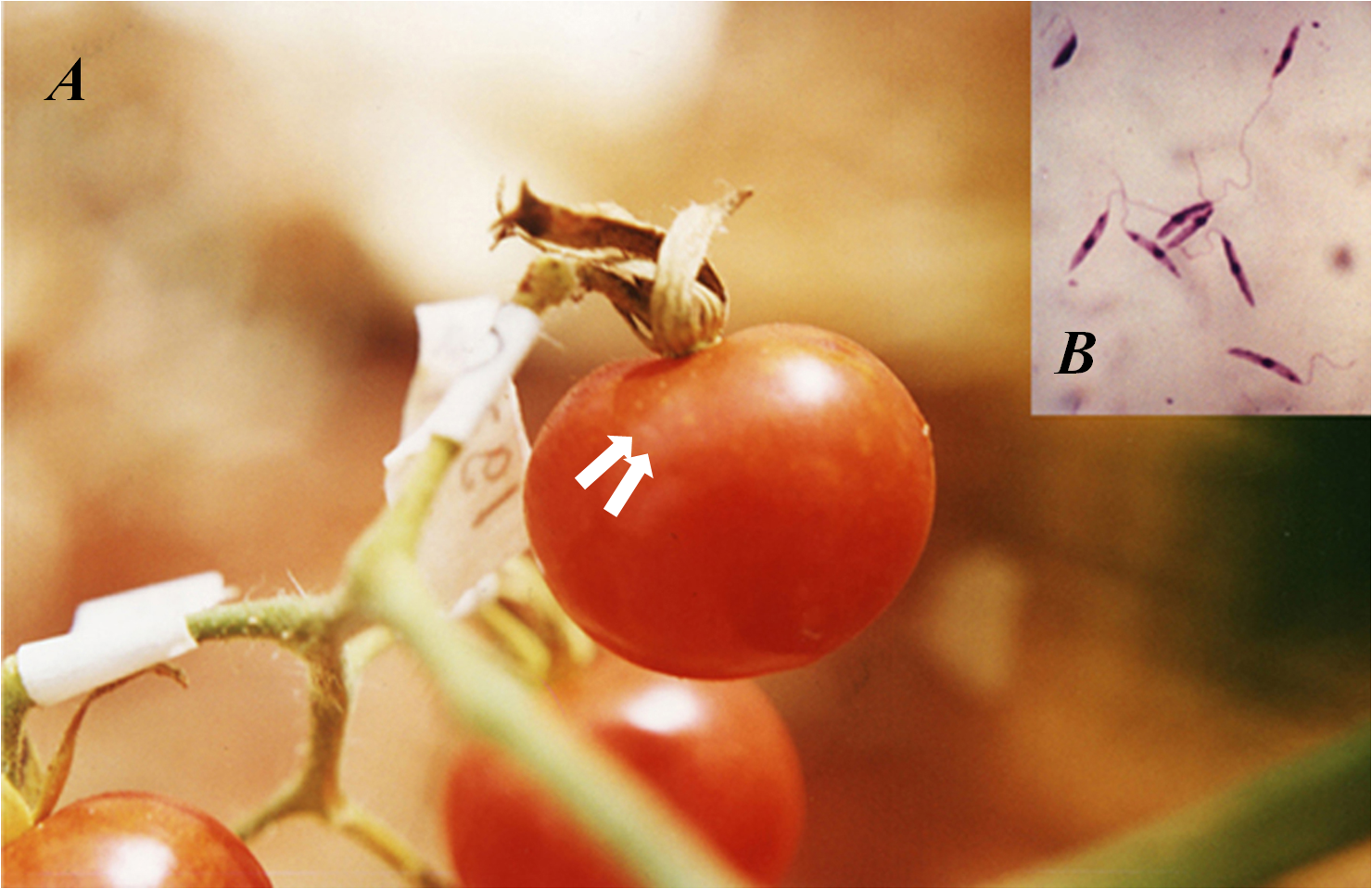

Supplement: Figure S1 — Tomatoes ( Lycopersicum esculentum ) infected with Phytomonas serpens . A and B . (A) Tomatoes infected. (B) Living culture flagellates forms of P. serpens (original magnification 400 X). Arrows indicate local infection on the fruit. (TIF) [file pone.0068299.s001.tif]

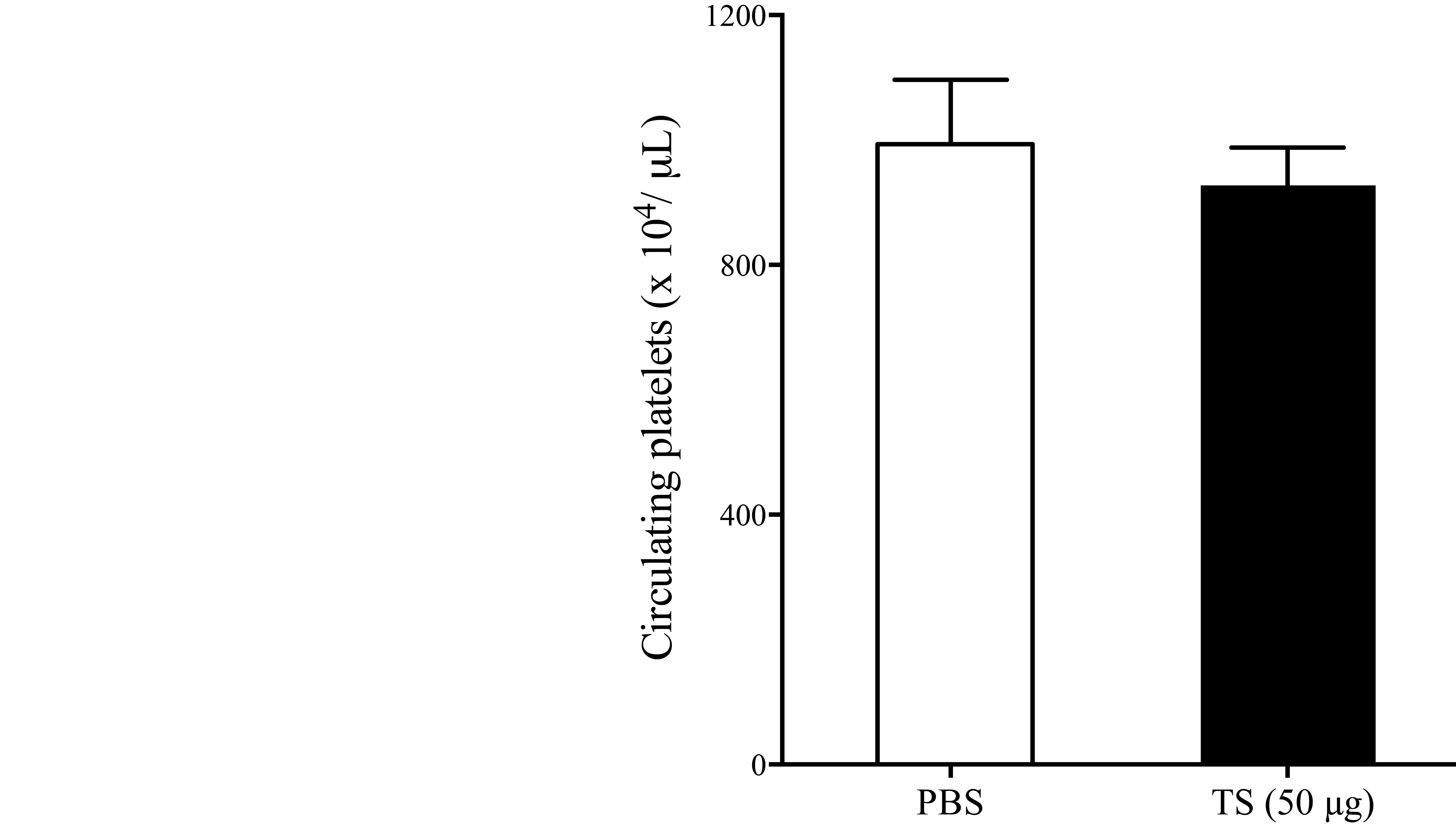

Supplement: Figure S2 — Heat-inactivated TS does not modify the life span of platelets. C57BL/6 mice were inoculated i.p. with 50 µg of recombinant TS heat-inactivated. Platelets counts were determined 24 h later. Values represent the mean ± standard error and are representative of two independent experiments; using 4 mice per group Results were analyzed by analysis of variance (ANOVA) followed by Bonferroni multiple comparisons test. Asterisks indicate significant differences (p<0.05) when compared with control group (PBS). (TIF) [file pone.0068299.s002.tif]

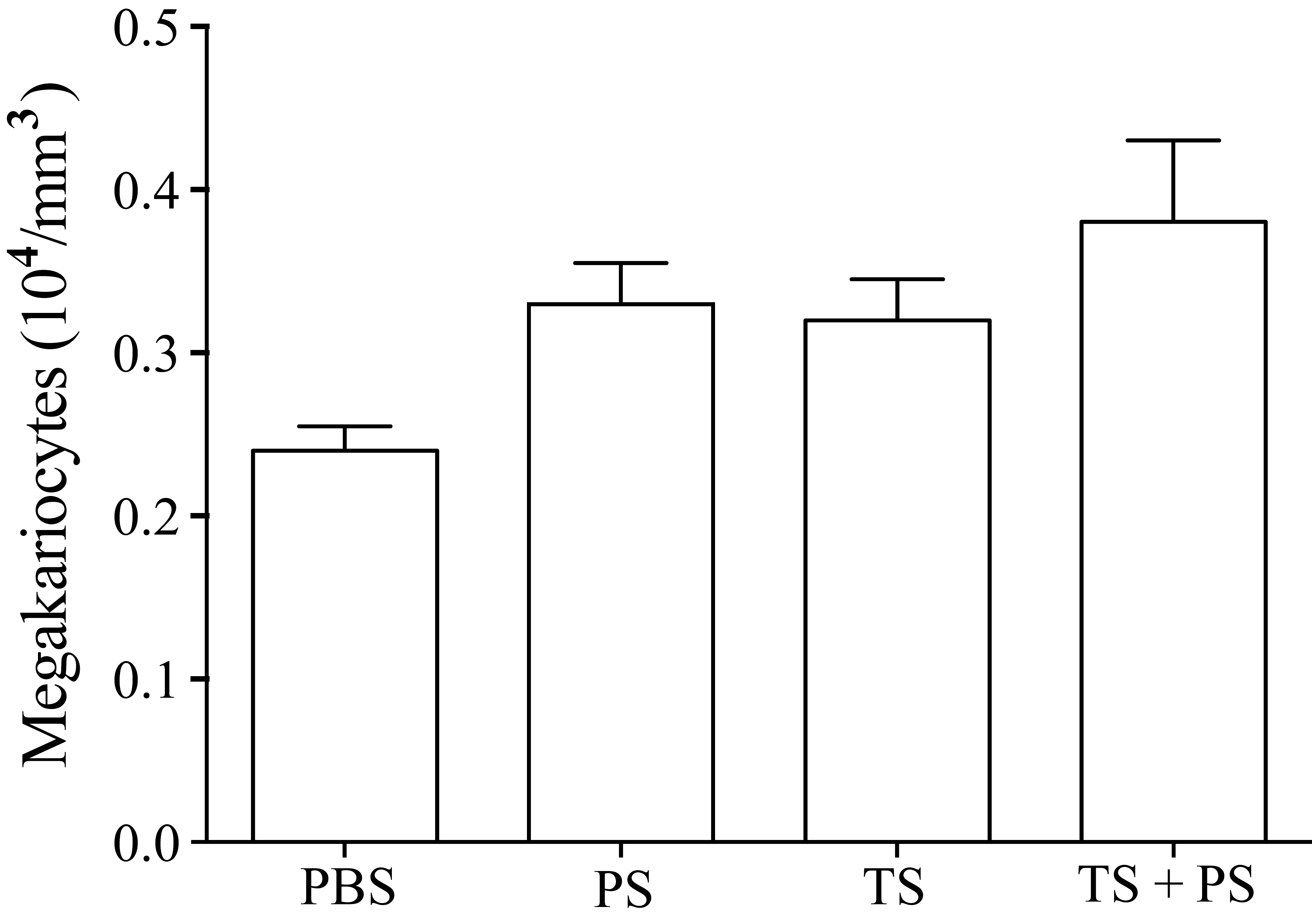

Supplement: Figure S3 — Oral exposure to P. serpens does not induce alterations in megakariocyte counts. The mice received by gavage 2×10^8^ living P. serpens parasites four times at weekly intervals and an i.p. 1 week later the mice were inoculated i.p. with 50 µg of recombinant TS. Megakariocyte counts were determined 24 h later. Values represent the mean ± standard error and are representative of two independent experiments, using 12 mice per group. Results were analyzed by analysis of variance (ANOVA) followed by Bonferroni multiple comparisons test. PBS (phosphate-buffered saline, pH 7.2) and PS (immunized with P. serpens). (TIF) [file pone.0068299.s003.tif]
